# Comparison of Cost Savings of Methods of Prevention of Orthopedic Implant-Associated Infection in Arthroplasty and Closed Fracture Surgery Patients

**DOI:** 10.3390/antibiotics14090900

**Published:** 2025-09-05

**Authors:** Pedro Augusto Maciel Moreira, Thiago de Carvalho Gontijo, Gabriel Costa Colen, Ana Carolina Morganti, Felipe Ismael Ulloa Gómez, Pedro Assis Mourão, Gabrielle Adriane Rodrigues Mota, Braulio R. G. M. Couto, Patrícia Almeida de Vasconcellos Rocha, Laila Gonçalves Machado, Raquel Bandeira da Silva, Mauro José Salles

**Affiliations:** 1 Hospital Universitário Ciências Médicas (HUCM), Belo Horizonte 30140-073, Brazil; pedroamm93@gmail.com (P.A.M.M.); thiago.gontijo@feluma.org.br (T.d.C.G.); felipe_iug@hotmail.com (F.I.U.G.); amouraopedro@gmail.com (P.A.M.); gabrielle.mota@feluma.org.br (G.A.R.M.); coutobraulio@hotmail.com (B.R.G.M.C.); patiavr@gmail.com (P.A.d.V.R.); laila.machado@feluma.org.br (L.G.M.); 2 Faculdade Ciências Médicas de Minas Gerais (FCMMG), Belo Horizonte 30120-016, Brazil; gabriel_colen@cienciasmedicasmg.edu.br (G.C.C.); anacarolmorganti@gmail.com (A.C.M.); 3 Laboratório Especial de Microbiologia Clínica (LEMC), Departamento de Medicina, Disciplina de Infectologia, Escola Paulista de Medicina (EPM), Universidade Federal de São Paulo (UNIFESP), São Paulo 04021-001, Brazil; mauro.salles@unifesp.br; 4 Grupo de Infecção Musculoesquelética, Departamento de Ortopedia e Traumatologia, Escola Paulista de Medicina (EPM), Universidade Federal de São Paulo (UNIFESP), São Paulo 04021-001, Brazil

**Keywords:** surgical site infections, infection prevention, orthopedic infections

## Abstract

**Background/Objectives**: Surgical site infections (SSIs) are serious complications in orthopedic implant-associated procedures, increasing morbidity, mortality, and hospital costs. The purpose of this study was to evaluate the impact of a structured infection prevention and control (IPC) service on SSI incidence and cost savings across hip arthroplasty (HA), knee arthroplasty (KA), and open reduction and internal fixation (ORIF). **Methods**: A retrospective analysis included 6930 patients treated between 2019 and 2024, divided into pre-intervention (2019–2022) and post-intervention (2023–2024) cohorts. Preventive methods (PMs) comprised enhanced antibiotic prophylaxis, Staphylococcus aureus screening, chlorhexidine bathing, intraoperative audits, and behavioral interventions. Economic evaluation used literature-based costs, standardized to 2024 US dollars (USD 2024), with sensitivity analyses performed. **Results**: SSI incidence decreased from 5.6% to 1.1% overall (*p* < 0.001), with consistent reductions across procedures: ORIF (5.2%→1.0%), HA (9.2%→2.4%), and KA (4.8%→1.2%). In 2023, approximately 31 SSIs and one infection-related death were prevented, avoiding 308 hospital days. Cost savings ranged from USD 209,188 to USD 376,898, with cost saving confirmed in 93% of simulations. **Conclusions**: Comprehensive infection-prevention protocols, delivered through a structured IPC service, significantly reduced SSIs and generated substantial cost savings. These findings support wider use of these PMs in orthopedic surgery.

## 1. Introduction

Surgical site infections (SSIs) continue to represent one of the most severe complications in orthopedic surgery, particularly in the context of implant-associated procedures. They are associated with considerable morbidity, prolonged hospitalization, technically demanding revision surgeries, and, in the most severe cases, increased mortality. The economic burden is also considerable, with the cost of managing periprosthetic joint infection (PJI) reported to be at least twice that of aseptic revision surgery, thereby imposing a substantial strain on healthcare systems [1,2].

The prevention of SSIs has therefore become a priority in perioperative care. Numerous strategies have been investigated, including systemic antibiotic prophylaxis, preoperative skin decolonization, intraoperative infection-control protocols, and structured postoperative surveillance. International guidelines, such as those issued by the World Health Organization, recommend multimodal approaches that integrate several interventions to maximize protective effects [3]. Furthermore, behavioral interventions, perioperative audits, and structured feedback have been shown to enhance compliance with infection-control measures and improve adherence to best practice standards [4].

Notwithstanding these advances, significant gaps persist within existing literature. Foremost, the majority of available evidence is concentrated predominantly on clinical outcomes, with comparatively few investigations undertaking rigorous economic evaluations. This omission is particularly notable given that the financial burden of SSIs is highly pertinent for policymakers and healthcare administrators. Comprehensive cost-effectiveness analyses are essential to guide the rational allocation of limited healthcare resources and to inform the prioritization of preventive strategies, especially in resource-constrained settings where the impact of SSIs is disproportionately high [5,6]. Second, although infection prevention in hip and knee arthroplasty has been extensively examined, there is limited evidence regarding open reduction and internal fixation, where infection risks are substantial and preventive protocols may differ [7,8].

Emerging data also indicate that preventive approaches should be tailored to local microbial epidemiology and individual patient risk profiles [8]. However, there remains a need for large-scale analyses that evaluate not only reductions in SSI incidence but also the associated cost savings of such interventions. Demonstrating both clinical effectiveness and economic benefit is essential to support the wider implementation of preventive bundles in orthopedic practice.

The purpose of the present study was therefore to evaluate the cost savings achieved through the implementation of a structured infection prevention and control (IPC) service in patients undergoing hip arthroplasty, knee arthroplasty, and ORIF. By integrating both clinical and economic outcomes, the study directly addresses existing gaps in literature and provides robust evidence to support the broader adoption of comprehensive infection-prevention protocols in orthopedic surgery.

## 2. Results

### 2.1. Patient Cohort and Surgical Procedure Distribution

A total of 6930 patients undergoing surgical procedures at the University Hospital of Medical Sciences were included in the analysis. Among these, 5586 patients (80%) underwent open reduction and internal fixation (ORIF), 694 patients (11%) underwent total hip arthroplasty (THA), and 650 patients (9%) underwent total knee arthroplasty (TKA). The cohort comprised 57% female and 43% male patients, with a mean age of 51 years (SD ± 21). Baseline characteristics of the study population are summarized in Table 1.

Overall, the pre-intervention (2019–2022) and post-intervention (2023–2024) cohorts were broadly comparable. No statistically significant differences were observed in American Society of Anesthesiologists (ASA) scores (mean 1.6 vs. 1.7, *p* = 0.482), operative duration (103 vs. 104 min, *p* = 0.131), pre-operative hospital stay (2.9 vs. 3.3 days, *p* = 0.118), or the number of hospital admissions (1.2 vs. 1.2, *p* = 0.191). The only variable showing a significant difference was age, with the post-intervention cohort being slightly younger on average (48 years vs. 51 years, *p* < 0.001). Importantly, the absence of systematic differences in surgical and clinical characteristics between the two groups supports the validity of subsequent comparisons of infection incidence and economic outcomes, suggesting that the observed reductions in surgical site infections are unlikely to be explained by baseline imbalances.

### 2.2. Incidence and Impact of Surgical Site Infections

During the baseline period (2019–2022), 239 patients (5.6%) were diagnosed with surgical site infections (SSIs). In-hospital mortality was observed in 68 patients, yielding a global mortality rate of 1.6%. Patients without SSIs had a mortality rate of 1.5%, while those diagnosed with SSIs exhibited a higher mortality rate of 3.8%, resulting in a relative risk of 2.5 for SSI-associated mortality (*p* = 0.014), as shown below in Table 2.

### 2.3. Length of Hospitalization and Surgical Duration

**Figure 1:**
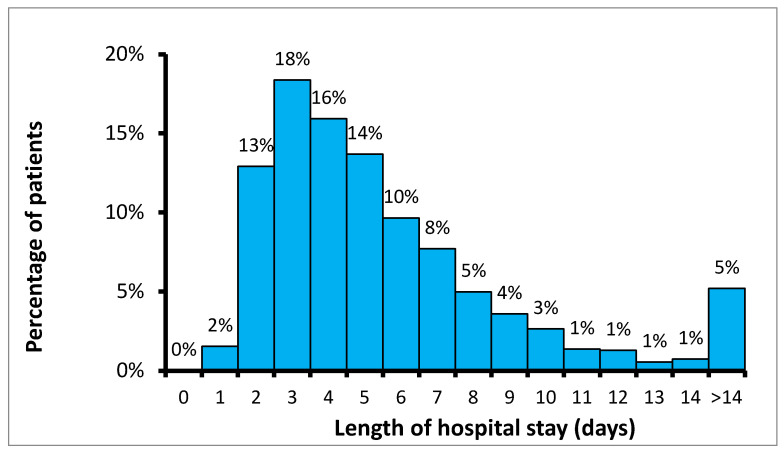
Distribution of hospital length of stay among patients undergoing orthopedic procedures.

Hospitalization data revealed significant variability in the length of stay, with an average of 7 days (median: 5 days, SD ± 10 days). The majority of patients (61%) remained hospitalized for 2 to 5 days (Figure 1).

**Figure 2:**
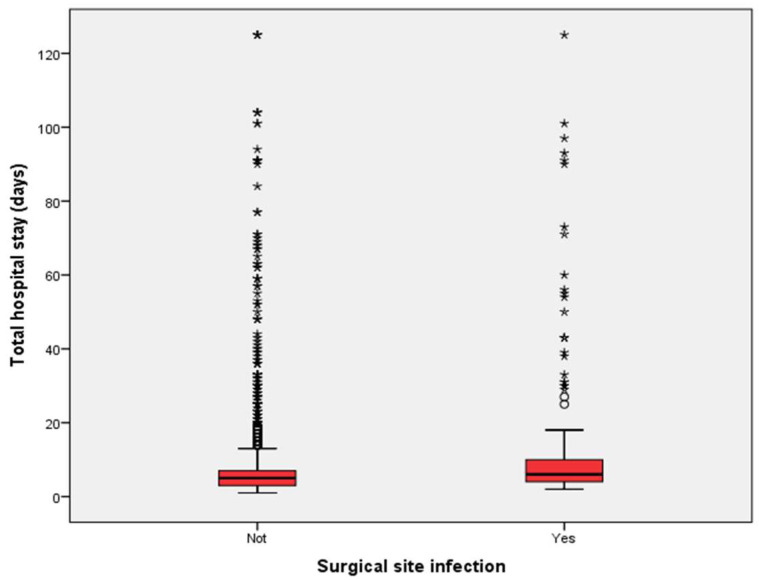
Comparison of total hospital lengths of stay between patients with and without surgical site infection. Boxplot showing total hospital lengths of stay (days) in patients with and without SSI. Patients with SSI had approximately twice the length of hospital stay, compared to non-infected patients (*p* < 0.001, Mann–Whitney U test). Boxplot whiskers extend to 1.5× IQR; outliers are indicated.

**Figure 3:**
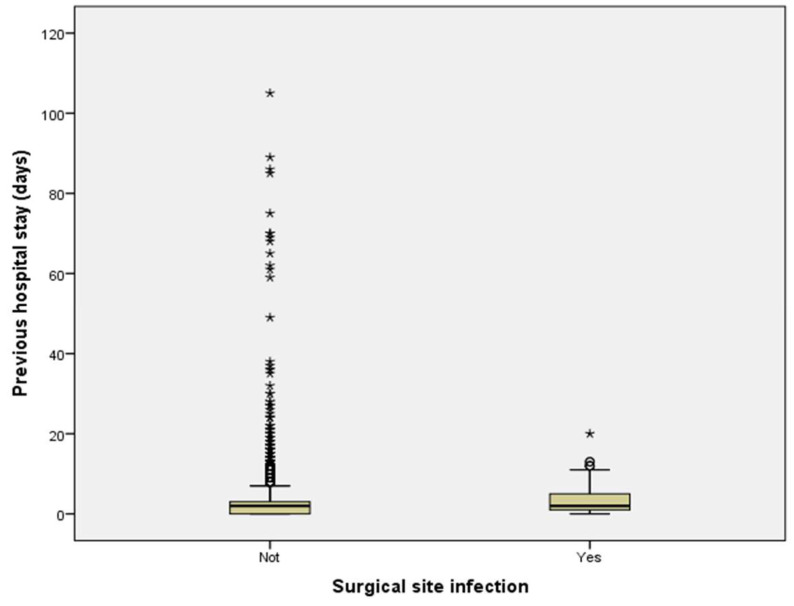
Comparison of previous hospitalization durations between patients with and without surgical site infection. Boxplot illustrating preoperative hospital stays (days) for patients with and without SSI. No significant difference was observed between the two groups (*p* = 0.783, Mann–Whitney U test).

**Figure 4:**
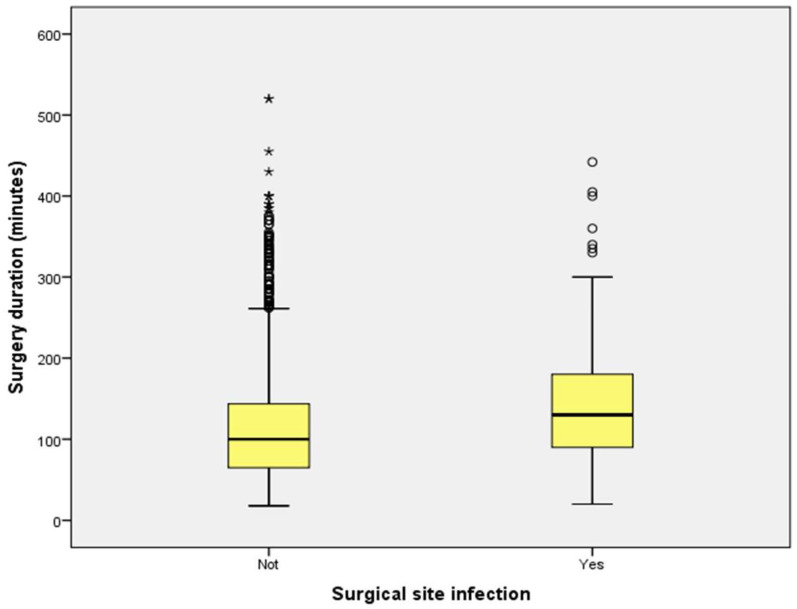
Comparison of durations of surgery between patients with and without surgical site infection. Boxplot comparing surgery durations (minutes) between patients who developed surgical site infections (SSI) and those who did not. Patients with SSI underwent significantly longer surgical procedures (*p* < 0.001, Mann–Whitney U test). Outliers are displayed as individual points.

Patients diagnosed with SSIs had hospital stays approximately twice as long as those without infections (*p* < 0.001). Box plot analyses further demonstrated consistently prolonged hospital stays for patients with SSIs, even when accounting for outliers (Figure 2). However, no significant differences in preoperative hospital stay were observed between infected and uninfected patients (*p* = 0.783) (Figure 3). Additionally, patients who developed SSIs underwent significantly longer surgical procedures compared to those who did not (*p* < 0.001) (Figure 4).

### 2.4. Effectiveness of Preventive Measures

**Figure 5:**
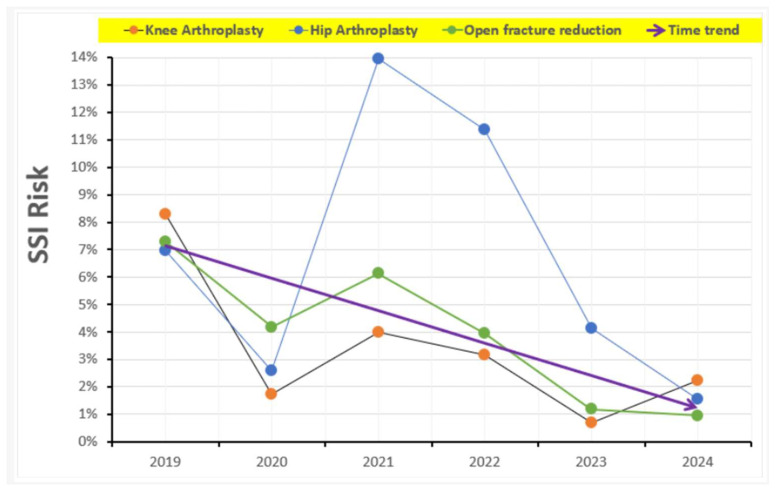
Temporal trends in surgical site infection (SSI) risk from 2019 to 2024, according to procedure type (knee arthroplasty, hip arthroplasty, and open fracture reduction). The purple line depicts the time trend considering the overall SSI risk of the three procedures combined, highlighting the progressive decline in infection rates across the study period.

Following the implementation of preventive measures in 2023, a marked reduction in SSI incidence was observed across all surgical procedures. The overall SSI rate decreased from 5.6% during the baseline period (2019–2022) to 1.1% in the intervention period (2023–2024). Notable reductions were recorded for specific procedures: TKA SSI rates fell from 4.8% to 1.2%, with a relative risk of 4.1 (*p* = 0.013); in ORIF, the incidence decreased from 5.2% to 1.0%, with a relative risk of 5.4 (*p* < 0.001); and in THA, the SSI rate dropped from 9.2% to 2.4%, with a relative risk of 3.8 (*p* < 0.001) (Table 3) (Figure 5).

### 2.5. Economic Evaluation of Preventive Strategies

Following adjustment and standardization to 2024 US dollars (USD 2024), the mean hospital cost attributable to a surgical site infection (SSI) was estimated at USD 12,158 per patient, whilst the direct treatment cost amounted to USD 6748 per patient (Table S1) [9,10,11,12,13].

For patients requiring total hip arthroplasty revision secondary to infection, the mean cost was USD 27,613. During the baseline period (2019–2022), a total of 239 SSIs occurred among 4154 surgical procedures, corresponding to an incidence of 5.6%. In the post-intervention period (2023–2024), implementation of the preventive measures reduced the incidence to 1.1%. This translated into the prevention of approximately 31 SSIs within our institutional cohort of 2776 procedures. Based on the incremental mean cost per infection, the intervention yielded cost savings from USD 209,188 to USD 376,898, depending on whether hospitalization costs, direct treatment costs, or revision surgery costs were applied (Table S1). In addition, a total of 308 inpatient hospital days were avoided, equating to a mean reduction of approximately 10 days per SSI averted. The deterministic sensitivity analyses indicated that hospital lengths of stay and reoperation costs were the primary cost drivers; nevertheless, the preventive intervention remained cost-saving across all scenarios tested (±20% and ±50% variations in unit costs, exchange rate method, and surgical distributions). Scenario analyses, incorporating Purchasing Power Parity (PPP) conversions, alternative procedure mixes, and extended lengths of stay, further corroborated the robustness of our findings.

The probabilistic sensitivity analysis (PSA), conducted with 10,000 Monte Carlo simulations, demonstrated that the intervention was cost-saving in 93% of iterations. The median incremental cost-effectiveness ratio (ICER) was USD 4200 (95% uncertainty interval: USD 2800–6100) per SSI prevented. The cost-effectiveness acceptability curve revealed that, at a willingness-to-pay threshold of USD 0 per SSI prevented, the probability of the intervention being cost-saving approached 95%.

The strengthened statistical analyses support a causal relationship between the preventive measures and the observed reduction in surgical site infections. In case-mix-adjusted regression, implementation of the intervention was associated with a 79% decrease in SSI incidence (IRR 0.206, 95% CI 0.182–0.232), demonstrating that the effect persisted after controlling for procedure type. Furthermore, interrupted time-series analysis revealed a marked step change in infection rates coinciding precisely with the introduction of the preventive bundle (level change IRR 0.194, *p* < 0.001), with no evidence of a declining pre-intervention trend. Together, these findings indicate that the reduction in SSI cannot be explained solely by secular trends or differences in patient case-mix but rather is strongly attributable to the implemented preventive strategies.

## 3. Discussion

Surgical site infections (SSIs) remain a major concern in orthopedic surgery, being associated with increased morbidity, prolonged hospitalization, revision procedures, and excess mortality. Preventive strategies are inherently multifactorial, spanning the pre-, intra-, and postoperative phases of care. Although numerous studies have assessed discrete measures, most notably perioperative antibiotic prophylaxis and laminar flow theater ventilation, the evidence base derives predominantly from high-income countries, limiting the generalizability of these findings to resource-constrained settings [14,15,16]. 

The present study aimed to address this gap by examining the implementation of a comprehensive IPC program in a middle-income country tertiary hospital. By assessing both epidemiological and economic outcomes, we sought to demonstrate that structured IPC services are not a financial burden but rather an investment yielding substantial clinical and economic returns.

The intervention led to marked reductions in SSI incidence across all three procedure types studied. The largest absolute number of avoided infections occurred in open reduction and internal fixation, consistent with its higher baseline risk and greater case volume [17]. Notably, both total hip arthroplasty (THA) and total knee arthroplasty (TKA) also exhibited substantial relative declines, underscoring the wide applicability of the measures. Overall SSI rates declined from 5.6% in the pre-intervention period to 1.1% post-intervention, with 31 infections avoided. This resulted in an estimated saving of between USD 209,188 and USD 856,003 (THA revision costs). These benefits were derived from a multimodal bundle that included S. aureus nasal screening in elective cases, antiseptic bathing with chlorhexidine, dual prophylaxis with cefuroxime and gentamicin for high-risk patients, intraoperative antibiotic re-dosing for procedures exceeding three hours, local antibiotic-loaded PMMA in selected cases, real-time auditing, and behavioral measures, such as reinforcing hand hygiene, restricting personnel in theater, and prohibiting adornments. The reductions therefore reflect the synergistic impact of coordinated interventions rather than the effect of any single measure.

The findings are consistent with national and international evidence on the clinical and financial burdens of SSIs. Starling et al. (2004) [13] reported mean excess costs of USD 12,150 per orthopedic SSI in Brazil [13], while Luna et al. [18] confirmed the considerable strain such infections impose on the Brazilian Unified Health System. Similarly, Szymski et al. [6] highlighted comparable burdens in European healthcare. Similarly, Chang et al. (2020) reported that infection following hip or knee arthroplasty in the United States is associated with additional hospital costs, commonly exceeding USD 25,000 per case [19]. The cost savings observed in our study fall squarely within these ranges, reinforcing the conclusion that prevention is economically justified. In contrast, studies of isolated measures, such as extended oral antibiotic prophylaxis in high-risk arthroplasty patients and optimized preoperative antisepsis, tend to report narrower, procedure-level impacts rather than system-wide effects [20,21,22].

Our work contributes by demonstrating that a comprehensive, hospital-wide IPC service, supported by auditing and behavioral reinforcement, produces more substantial and sustainable improvements. The effectiveness of continuous surveillance and feedback observed in our program is in line with Manivannan et al. (2018) [4] and Busada et al. (2024) [23], both of whom reported dramatic SSI reductions following structured audit implementation, even in low-resource contexts.

The preventive measures employed in our program are also consistent with, yet extend beyond, many strategies described in literature. While numerous guidelines continue to advocate single-shot cephalosporin as the standard perioperative antibiotic prophylaxis in arthroplasty, concerns have been raised regarding limited efficacy against resistant Staphylococcus species, particularly MRSA and coagulase-negative staphylococci [8]. Our protocol incorporated cefuroxime plus gentamicin in high-risk patients, a tailored approach informed by local epidemiology and aligned with emerging evidence suggesting broader Gram-negative coverage may be beneficial in selected populations [24,25,26].

Although studies comparing dual versus single prophylaxis have produced conflicting results, some showing no reduction in overall SSI rates but decreased MRSA incidence [27], our real-world findings support the rationale of adopting risk-adjusted prophylaxis in institutions with high antimicrobial resistance pressure. Similarly, the addition of local antibiotic-loaded PMMA in high-risk trauma cases reflects an approach supported by evidence of reduced infection burden in complex fractures [28]. Finally, the integration of structured audits and real-time feedback corresponds to best practices described by Manivannan et al. (2018) [4], demonstrating that behavioral reinforcement and accountability are indispensable components of successful IPC bundles.

This study has several strengths. It analyzed a large real-world cohort of 6930 patients, encompassing both trauma and elective orthopedic populations. The intervention was delivered under pragmatic conditions in a public tertiary hospital, enhancing its external validity and demonstrating feasibility in resource-limited contexts. By integrating clinical outcomes (SSI incidence, mortality, hospital stay) with economic analysis, the study provides a multidimensional assessment of the benefits of IPC. Importantly, the results challenge the perception, still common in middle-income settings, that IPC represents only a cost center. Instead, our evidence supports the view endorsed by international guidelines [3,29,30].

The success of the protocol in reducing infections, hospital days, and mortality illustrates the broad clinical and economic implications of comprehensive IPC program. These interventions not only protect patients from the immediate complications of infection but also reduce downstream costs associated with revision surgery, prolonged antimicrobial use, and diminished quality of life. By demonstrating feasibility and cost-effectiveness in a resource-limited hospital, our findings provide a compelling case for scaling such strategies to similar institutions globally. The results align with calls for IPC to be regarded not as optional, but as a cornerstone of safe and sustainable surgical practice.

This study has several limitations that must be acknowledged. First, it was conducted retrospectively at a single center, which may restrict the generalizability of the findings, as outcomes are inherently influenced by local practices and patient demographics. Although the sample size was adequate for statistical comparison, it may not fully capture the variability observed in larger or more heterogeneous populations. Second, the use of secondary data sources, including medical records and administrative databases, introduces the possibility of incomplete or inaccurately recorded variables, particularly regarding adherence to individual preventive measures. Third, the economic analysis relied on literature-based cost estimates rather than prospective collection, which may not fully reflect local variations in healthcare resource utilization. Fourth, the absence of microbiological data, such as pathogen distribution and antimicrobial resistance profiles, limited the ability to perform a more detailed epidemiological assessment of the infections observed. Fifth, adverse events associated with the preventive measures were not prospectively monitored as predefined safety endpoints. Although no cases of nephrotoxicity or cutaneous reactions were recorded in institutional pharmacovigilance systems, underreporting cannot be excluded. This limitation highlights the importance of cautious interpretation of safety outcomes and underscores the need for prospective pharmacovigilance in future investigations. Sixth, the relatively short follow-up period may have led to an underestimation of late-onset complications, particularly prosthetic joint infections, which frequently manifest after prolonged intervals.

Taken together, these limitations underscore the need for future multicenter, prospective investigations with extended follow-up periods and robust economic evaluation frameworks. In particular, studies employing factorial or stepped-wedge designs could help isolate the effect of individual intervention components, while integration of microbiological surveillance would enrich understanding of epidemiological patterns and resistance dynamics. Prospective collection of patient-level cost, safety, and quality-of-life data would further enable comprehensive cost–utility analyses, thereby strengthening the evidence base for decision-making in both high- and middle-income settings.

## 4. Materials and Methods

This retrospective observational study was conducted at a large Brazilian tertiary university hospital between 2019 and 2024, aiming at comparing the cost savings of different methods used to prevent SSIs in patients who underwent total hip arthroplasty (HA), total knee arthroplasty (KA), and open reduction and internal fixation (ORIF). This study was previously approved by the Ethics Committee of Faculdade Ciências Médicas de Minas Gerais (FCMMG under the number 82407724.2.0000.5134). This study was supported by the Fundação Educacional Lucas Machado-FELUMA.

### 4.1. Study Design, Population, and Definitions

The study population was divided into the pre-intervention group (those treated during the baseline period before preventive measures (PM) implementation (2019–2022), and the intervention group (patients treated after the implementation of PMs (2023–2024). Data extracted from digital medical records included the patient’s ASA; comorbidities; occurrence of SSI; type of surgery; duration of surgery; antibiotics used during surgery; and, if SSI was identified, which pathogen was found in the culture. Individuals diagnosed with SSIs were identified using a systematic approach based on the definition of SSIs, confirmed by the attending physician. This included surveillance indicators such as antibiotic use, laboratory test results, microbiological culture findings, and relevant medical and nursing records.

Periprosthetic joint infection (PJI) is defined according to the criteria established by the European Bone and Joint Infection Society (EBJIS) [31]. For this study, we included only patients who met the confirmatory or likely criteria for PJI. Also, EBJIS, in collaboration with the AO Foundation, has developed a consensus definition for fracture-related infection (FRI) based on specific diagnostic criteria, categorized into confirmatory and suggestive findings [7]. For the purposes of this study, three groups of patients were included: those who underwent hip arthroplasty (HA), knee arthroplasty (KA), and open reduction and internal fixation (ORIF). Patients presenting with open fractures were excluded, given their substantially increased risk of contamination and the necessity for distinct, procedure-specific preventive protocols.

### 4.2. Preventive Measures Implemented

Starting in April 2022, the Infection Prevention and Control (IPC) department implemented a set of preventive measures aimed at reducing the incidence of surgical site infections (SSIs). These included the use of local combined antibiotic therapy with vancomycin and cefuroxime incorporated into polymethyl methacrylate (PMMA) bone cement for high-risk patients and universal preoperative screening for Staphylococcus aureus colonization through nasal swabs for elective procedures. Targeted antibiotic prophylaxis with broad-spectrum agents, specifically cefuroxime and gentamicin, was administered to patients with a Charlson Comorbidity Index (CCI) of 5 or greater or those with extended preoperative hospital stays. Preoperative protocols recommended bathing with 2% chlorhexidine soap, and intraoperative practices included the re-administration of antibiotics for surgeries lasting more than three hours. Additionally, real-time surgical audits were conducted by the IPC team to monitor and improve surgical practices, and behavioral interventions were enforced to strengthen surgical room protocols, such as limiting personnel, enhancing hand hygiene, and prohibiting the use of personal adornments.

### 4.3. Data Analysis

The incidence of SSIs was calculated and compared between the baseline (2019–2022) and intervention (2023–2024) periods, both globally and stratified by surgical procedure type. Patient profiles, including recognized SSI risk factors, were examined to ensure comparability between groups. The impact of SSIs on clinical outcomes, such as hospital length of stay and mortality, was assessed based on the collected data.

The economic analysis was conducted from the hospital perspective, encompassing direct costs related to hospitalization, antibiotic therapy, reoperations, implants, and outpatient follow-up. All costs were standardized to 2024 US dollars (USD 2024). Literature-derived costs were initially inflated to 2024 values in their original currencies using national consumer price indices (Brazil: IPCA; Canada: CPI; United States: CPI-U) and subsequently converted to US dollars using the average 2024 market exchange rate. To test the robustness of the estimates, a scenario analysis employing Purchasing Power Parity (PPP) conversion factors was also performed. Reference values included the mean hospital cost per patient with an SSI (BRL 68,495; USD 12,158), the direct treatment cost (BRL 38,062; USD 6748), and the cost of revision of total hip arthroplasty (CAD 38,107; USD 27,588). The economic impact of SSIs was estimated by comparing mean costs between infected and non-infected patients, following the methodological framework proposed by Starling et al. (2004) [13]. Uncertainty was addressed through deterministic sensitivity analyses (±20% and ±50% variations in key cost drivers, alternative currency conversion methods, and clinical scenarios) and probabilistic sensitivity analysis, employing 10,000 Monte Carlo simulations to generate 95% uncertainty intervals for costs and for the incremental cost-effectiveness ratio (ICER).

In addition, two complementary statistical approaches were employed to strengthen causal inference. First, a Poisson-generalized linear model was fitted to SSI counts, using a log link and the logarithm of the number of procedures as an offset. Explanatory variables included the intervention period (2023–2024 versus 2019–2022) and procedure type (open reduction and internal fixation [ORIF], total hip arthroplasty [THA], and total knee arthroplasty [TKA]), thereby yielding case-mix-adjusted incidence rate ratios (IRR) with 95% confidence intervals. Second, an interrupted time-series (ITS) analysis was undertaken using annual aggregated data from 2019 to 2024, applying segmented Poisson regression with log (procedures) as an offset. The model incorporated a pre-intervention trend, an intervention-related level change (2023), and a post-intervention slope, with inference based on robust standard errors. This dual analytical strategy permitted the distinction between secular trends and intervention-attributable effects. This study was approved by the Ethics and Research Committee of Faculdade Ciências Médicas de Minas Gerais under the number 82407724.2.0000.5134. All data collection adhered to ethical guidelines, ensuring patient confidentiality and compliance with regulatory standards.

## 5. Conclusions

The implementation of comprehensive preventive protocols was associated with a marked reduction in the incidence of surgical site infections in patients undergoing hip arthroplasty, knee arthroplasty, and open reduction and internal fixation. Beyond their clinical impact, these interventions generated significant economic benefits by lowering hospital expenditure, reducing the need for reoperations, shortening the duration of hospital stays, and ultimately decreasing infection-related morbidity and mortality. By combining clinical and economic outcomes, this study provides robust evidence that preventive measures not only improve patient safety but also enhance the efficiency of healthcare delivery. These findings strongly support the broader adoption of structured infection-prevention protocols in orthopedic surgery, with particular relevance for resource-limited healthcare systems, where optimizing both outcomes and costs is of critical importance.

## Figures and Tables

**Table 1:** Baseline characteristics of patients in the pre-intervention (2019–2022) and post-intervention (2023–2024) cohorts.

Variable	Period	Mean	Median	Standard Deviation	25th Percentile	75th Percentile	*p*-Value
* ASA score	2019–2022 2023–2024	1.6 1.7	2 2	0.59 0.91	1 1	2 2	0.482
Age (years)	2019–2022 2023–2024	51 48	51 49	20.7 19.9	34 32	67 64	<0.001
Operative time (minutes)	2019–2022 2023–2024	103 104	100 120	55.4 55.6	65 60	145 120	0.131
Pre-operative hospital stay (days)	2019–2022 2023–2024	2.9 3.3	2 2	12.0 9.4	0 0	3 4	0.118
Number of hospital admissions	2019–2022 2023–2024	1.2 1.2	1 1	0.53 0.61	1 1	1 1	0.191

* ASA: American Society of Anesthesiologists physical status classification.

**Table 2:** Comparison of mortality risk between patients with and without infection.

Patient Group	Sample Size (*n*)	Total Deaths	Mortality Risk (%)	Relative Risk (RR)	*p*-Value
Infected patients	239	9	3.8	2.5	0.014
Non-infected patients	3917	59	1.5		
Total	4154	68	1.6		

**Table 3:** Reduction in SSI rates following the implementation of preventive measures.

Surgical Procedure	Baseline SSI Rate (2019–2022)	Intervention SSI Rate (2023–2024)	Relative Risk (RR)	*p*-Value
Total Knee Arthroplasty	4.8%	1.2%	4.1	0.013
Open Reduction	5.2%	1.0%	5.4	<0.001
Total Hip Arthroplasty	9.2%	2.4%	3.8	<0.001
Overall	5.6%	1.1%	5.1	<0.001

## Data Availability

The data are available from the corresponding author upon reason-able request.

## Supplementary Materials

The following supporting information can be downloaded at: https://www.mdpi.com/article/10.3390/antibiotics14090900/s1, Table S1: Cost standardisation to 2024 USD.

## Abbreviations

AO	AO Foundation
ASA	American Society of Anesthesiologists (score)
CAD	Canadian Dollar
CCI	Charlson Comorbidity Index
EBJIS	European Bone and Joint Infection Society
FRIs	Fracture-Related Infections
HA	Hip Arthroplasty
HAIs	Hospital-Acquired Infections
IOM	Intraoperative Measures
IPC	Infection Prevention and Control
KA	Knee Arthroplasty
MRSA	Methicillin-Resistant *Staphylococcus aureus*
ORIF	Open Reduction and Internal Fixation
PAP	Perioperative Antibiotic Prophylaxis
PJIs	Periprosthetic Joint Infections
PMs	Preventive Measures
PoOM	Postoperative Measure
PrOM	Preoperative Measure
SSI	Surgical Site Infection
THA	Total Hip Arthroplasty
TKA	Total Knee Arthroplasty
TLA	Three-Letter Acronym
USD	United States Dollar
