# Supplementary material for: Comparison of Cost Savings of Methods of Prevention of Orthopedic Implant-Associated Infection in Arthroplasty and Closed Fracture Surgery Patients
Source: Antibiotics (Basel). 2025 Sep 5;14(9):900. doi: 10.3390/antibiotics14090900 (PMC12466538; doi:10.3390/antibiotics14090900)
Supplement: Supplementary file 1 [file antibiotics-14-00900-s001.zip › antibiotics-3836300-supplementary.pdf]

Table S1 – Cost standardisation to 2024 USD

Source	Original cost (currency/year)	Inflation index applied	Value after inflation (local currency)	Conversion rate to USD 2024	Final cost (USD 2024)
Hospital cost per SSI patient (Brazil)	R$ 68,495 (BRL, 2017)	IPCA to 2024	R$ 84,500 (2024 BRL)	1 USD = 6.95 BRL (2024 avg)	USD 12,158
Direct treatment cost per SSI patient (Brazil)	R$ 38,062 (BRL, 2017)	IPCA to 2024	R$ 46,900 (2024 BRL)	1 USD = 6.95 BRL (2024 avg)	USD 6,748
Revision cost for THA (Canada)	CAN$ 38,107 (CAD, 2019)	CPI (Canada) to 2024	CAN$ 42,800 (2024 CAD)	1 USD = 1.55 CAD (2024 avg)	USD 27,613

Original costs in local currencies were adjusted to 2024 values using official inflation indices (IPCA for Brazil; CPI for Canada). Adjusted costs were then converted to USD based on 2024 average exchange rates (1 USD = 6.95 BRL; 1 USD = 1.55 CAD). SSI = *surgical site infection*; THA = *total hip arthroplasty*.

References:

1. Brazilian Institute of Geography and Statistics (IBGE). National Consumer Price Index (IPCA) [Internet]. Rio de Janeiro: IBGE; [cited 2025 Aug 25]. Available from: https://www.ibge.gov.br

2. Statistics Canada. Consumer Price Index (CPI) [Internet]. Ottawa: Statistics Canada; [cited 2025 Aug 25]. Available from: https://www.statcan.gc.ca

3. U.S. Bureau of Labor Statistics. Consumer Price Index (CPI-U) [Internet]. Washington (DC): U.S. Bureau of Labor Statistics; [cited 2025 Aug 25]. Available from: https://www.bls.gov

4. World Bank. Official exchange rates and Purchasing Power Parity (PPP) conversion factors [Internet]. Washington (DC): World Bank; [cited 2025 Aug 25]. Available from: https://data.worldbank.org

5. Starling, C.E.F.; Couto, B.R.G.M.; Fialho, A.S.; Alves, A.A. Impacto das infecções hospitalares na lucratividade de hospitais privados brasileiros. *Prática. Hosp. Urgências.* **2004**, *34*, 77–78.
